# Eradication of Drug-Tolerant *Mycobacterium tuberculosis* 2022: Where We Stand

**DOI:** 10.3390/microorganisms11061511

**Published:** 2023-06-06

**Authors:** Alessio Lanni, Angelo Iacobino, Lanfranco Fattorini, Federico Giannoni

**Affiliations:** Department of Infectious Diseases, Istituto Superiore di Sanità, Via Regina Elena 299, 00161 Rome, Italy; alessio.lanni@guest.iss.it (A.L.); angelo.iacobino@iss.it (A.I.); lanfranco.fattorini@iss.it (L.F.)

**Keywords:** *Mycobacterium tuberculosis*, tuberculosis, drug-resistance, drug combinations, drug-tolerance, persisters, differentially detectable cells, mice models, tuberculosis therapy, clinical trials

## Abstract

The lungs of tuberculosis (TB) patients contain a spectrum of granulomatous lesions, ranging from solid and well-vascularized cellular granulomas to avascular caseous granulomas. In solid granulomas, current therapy kills actively replicating (AR) intracellular bacilli, while in low-vascularized caseous granulomas the low-oxygen tension stimulates aerobic and microaerophilic AR bacilli to transit into non-replicating (NR), drug-tolerant and extracellular stages. These stages, which do not have genetic mutations and are often referred to as persisters, are difficult to eradicate due to low drug penetration inside the caseum and mycobacterial cell walls. The sputum of TB patients also contains viable bacilli called differentially detectable (DD) cells that, unlike persisters, grow in liquid, but not in solid media. This review provides a comprehensive update on drug combinations killing in vitro AR and drug-tolerant bacilli (persisters and DD cells), and sterilizing *Mycobacterium tuberculosis*-infected BALB/c and caseum-forming C3HeB/FeJ mice. These observations have been important for testing new drug combinations in noninferiority clinical trials, in order to shorten the duration of current regimens against TB. In 2022, the World Health Organization, following the results of one of these trials, supported the use of a 4-month regimen for the treatment of drug-susceptible TB as a possible alternative to the current 6-month regimen.

## 1. Introduction

*Mycobacterium tuberculosis* (Mtb) is the causative agent of tuberculosis (TB). The World Health Organization (WHO) estimated that in 2021 approximately 10.6 million people developed tuberculosis (TB), and about 1.6 million died from the active disease, making TB one of the leading causes of death worldwide [1]. Furthermore, an estimated 1.7 billion people have a latent TB infection (LTBI) [2]. The current antibiotic treatment of active, drug-susceptible (DS)-TB requires the administration of a combination therapy for 6 months, including the first-line drugs rifampin (R), isoniazid (H), ethambutol (E) and pyrazinamide (Z) (R-H-E-Z) for 2 months, followed by R and H for 4 months.

The lungs of patients with active TB and LTBI contain a spectrum of granulomatous lesions ranging from solid and well-vascularized cellular granulomas to avascular caseous granulomas [3,4,5]. In these lesions, heterogeneous subpopulations of Mtb cells, ranging from actively replicating (AR) to nonreplicating (NR) dormant stages, coexist. In solid granulomas, current therapy kills AR intracellular bacilli inside the macrophages, while in low-vascularized caseous granulomas, the low-oxygen tension stimulates aerobic and microaerophilic AR bacilli to transit into NR, hypoxic, drug-tolerant stages. Hypoxic bacilli use host triacylglycerol and cell wall mycolates to accumulate lipid droplets in lipid-loaded (foamy) macrophages [6,7,8]. These cells release lipid droplets into the hypoxic necrotic core of closed caseous granulomas, which contain extracellular, NR drug-tolerant, phenotypically drug-resistant bacilli. The eradication of these bacilli using current TB therapy is difficult due to the low penetration of the drugs inside the caseous granulomas and NR Mtb cell walls [4,9]. Tubercle bacilli surviving inside necrotic granulomas are often referred to as persisters and are thought to be responsible for the long duration of TB therapy [4]. The closed caseous granulomas expand and fuse with the structures of the bronchial tree, and they form cavities in which the caseum liquefies after coming into contact with the air. In the liquefied material, the NR cells rapidly multiply and are released into the airways as a mixture of AR and NR bacilli, which are detected in the sputum of pulmonary TB patients as colony-forming units (CFU) on solid media such us Middlebrook 7H10 agar plates and Löwenstein-Jensen slants [10,11].

However, the sputum of drug-untreated patients was found to also contain viable Mtb bacilli that were not detectable as CFU [12,13,14,15,16,17,18]. The number of these viable organisms in the sputum was estimated through a limiting dilution (LD) technique in a liquid medium, using the most probable number (LD-MPN) method. These subpopulations were called with various terms, including viable but nonculturable cells (VBNC), differentially culturable TB (DCTB) cells and—perhaps the most recently used term—differentially detectable (DD) cells [16]. Several other bacterial species were found to exist in a VBNC state [19]. When the number of Mtb CFU is subtracted from the number of Mtb cells obtained through the LD-MPN method, the difference is the DD Mtb number [16]. Tubercle bacilli growing in liquid but not on solid media were also found in chronic, murine TB [20]. Some studies showed that DD cells decreased less rapidly than CFU after the initiation of TB treatment, suggesting that they are also drug-tolerant cells [15,18].

Overall, these observations on dormant, drug-tolerant bacilli (persister cells and DD cells) may aid in finding new drugs and in designing shorter drug combination regimens. The goal of this review is to give an update on the reported in vitro and in vivo combinations eradicating all dormant Mtb stages (persister cells and DD cells), and on their impact on the therapeutic strategies endorsed by the WHO to shorten the microbiological and therapeutical management of DS- and drug-resistant (DR)-TB.

## 2. Persister Cells

### 2.1. Overview

Persister cells (persisters) were reported in several Gram-positive and Gram-negative bacteria [21] and in many eukaryotes, including yeasts (*Candida* spp.) [22], and protozoa (*Plasmodium*, *Leishmania*, *Trypanosoma* and *Toxoplasma* spp.) [23], where they contribute to establish persistent infections and cause treatment failures, without genetic drug resistance. Persisters were also described in human cancer, where they survive chemotherapy and promote the development of drug resistance [24,25]. All investigators studying persisters uniformly highlight that the eradication of persisters could lead to a strong reduction in treatment failures and relapses in several clinical settings. The underlying reasons for the development of persisters seem to be multifactorial, involving non-genetic transient mechanisms switched on in microorganisms and cancer cells, as well as sub-optimal pharmacodynamics and immune responses in the host.

### 2.2. Bacterial Persisters

The term “persisters” was used for the first time in 1944 by Joseph Bigger, who realized that penicillin failed to sterilize *Staphylococcus pyogenes* cultures due to the generation of a small number of cocci “individually insensitive to penicillin, but developing normally when penicillin is destroyed” [21,26,27]. Well in advance, he specified that “persisters are not resisters”, i.e., cells that we now call antibiotic-resistant mutants. Bigger also observed that “persisters are believed to be insensitive to penicillin because they are in a dormant, non-dividing phase”.

In the search for persisters genes, starting in the 1980s, Harris Moyed and coworkers isolated mutants of *Escherichia coli* with a vastly increased frequency of persistence (Hip mutants) [28]. The *hip* locus affects the frequency of persistence to the lethal consequences of selective inhibition of either DNA or peptidoglycan synthesis [29]. The *hipA7* strain produced about 1% persisters and, after exposure to ampicillin, it generated about 1000 times more persisters than the wild-type strain [27].

In 2019, Nathalie Balaban et al., spurred by increased concerns about antibiotic resistance and several papers published on the persistence phenomenon, released an international consensus statement on definitions and guidelines for research on antibiotic persistence [30]. Antibiotic resistance was defined as the ability of bacteria to replicate, —and not just survive—in the presence of a drug, with the minimum inhibitory concentration (MIC) being the most common measure of the resistance level. Resistance is inherited and may be acquired by genetic mutations or the horizontal gene transfer of resistance-encoding genes. Instead, antibiotic persistence is the ability of a subpopulation to survive exposure to a bactericidal concentration [30]. The hallmark of antibiotic persistence is the biphasic killing curve. When persisters regrow in the absence of antibiotics, they give rise to a population of drug-sensitive cells. The size of the persister subpopulation is related to the class of drug and not to the drug concentration. Persisters cannot replicate in the presence of a drug and are killed at a lower rate than the susceptible population from which they arise. The terms persistence and tolerance refer both to increased survival in the presence of an antibiotic, with no MIC increase. However, persistence refers to a cell subpopulation, while tolerance deals with the general ability of a population to survive longer drug treatments. Thus, persisters are a subpopulation of drug-tolerant bacteria, and tolerance and persistence may arise without dormancy [30,31]. The time required to kill these populations is quantified as the minimum duration for killing and is useful for comparing their survival fractions. The international consensus statement also showed that there are two kinds of persistence: type I, triggered by a stress signal (nutrient starvation, high cell number, acid stress, immune factors), and type II (spontaneous), which develops stochastically without any trigger, at a rate that is constant during growth [30,32,33,34]. The spontaneous and drug-induced persistence are difficult to distinguish and can be studied through direct observation of single bacterial cells using microfluidic devices [30,32,33]. Persistent bacterial infections cause the deaths of millions of people every year. In 2022, a bibliometric analysis of the top 100 cited studies on bacterial persisters was reported [35].

### 2.3. Mycobacterium tuberculosis Persisters

*M. tuberculosis* persisters are NR multidrug-tolerant bacilli genetically identical to drug-susceptible cells, which survive in the hostile environments of granulomas and in the sputum and adipose tissues of TB patients [36,37,38]. These cells are primarily involved in LTBI and TB relapses. Microenvironments with low oxygen tension and pH, nutrient starvation, reactive oxygen and nitrogen stresses, lead to the formation of reservoirs of phenotypically drug-resistant cells with reduced antibiotic uptake and increased drug efflux, which renders antibiotics ineffective without genetic resistance [9,36,39]. Responses against drug treatment of Mtb under hypoxic conditions and other stresses were modeled and/or reviewed in several papers [11,40,41,42,43,44,45].

The primary reason for the 6-month duration of the first-line TB therapy (R-H-E-Z) is due to the ability of Mtb to generate heterogeneous subpopulations adapting to different microenvironments within granulomas. The microfluidic devices for single-cell analysis revealed that the wide diversity of tubercle bacilli can be due to the fact that the mycobacterial cell division is asymmetrical, with the old pole elongating more than the new pole [46,47]. Asymmetric distribution of irreversibly oxidated proteins generates Mtb sister cells with different growth properties and drug susceptibility. In particular, sister cells with a low amount of these proteins are less sensitive to drugs, likely contributing to persisters formation. Asymmetric growth in mycobacteria is regulated mainly by the division protein Wag31 and the growth inhibitor LamA [46].

Other studies on single cells reported that Mtb persisters are rare low-energy cells formed stochastically during normal growth [48]. Stochastic variations may occur in the expression of an energy-generating component. Indeed, Mtb cells cultivated in a minimal medium with acetate showed cell-to-cell variations in the level of mRNA coding for the acetate kinase (AckA), a low-energy-generating mechanism. This led to the formation of persister cells with decreased AckA, which showed low ATP levels and inactive drug targets. The knowledge that *Staphylococcus aureus* and *E. coli* persisters also have low ATP levels is in keeping with these observations [49,50]. In this view, it was hypothesized that all the culture conditions leading to persistence converge in the inability to re-initiate a new round of DNA replication caused by an insufficient level of the initiator complex ATP-DnaA, resulting in the lack of the formation of a functional orisome [51]. Mycobacteria heterogeneity generated in part by stochastic processes may be amplified by innate asymmetric growth and division patterns [46].

Of importance, persister cells may generate genetically resistant mutants. Indeed, prolonged exposure to lethal concentrations of R or the fluoroquinolone moxifloxacin (M) induced the formation of Mtb and *Mycobacterium smegmatis* persisters, which carried elevated levels of the reactive oxygen species (ROS) hydroxyl radical, superoxide radical and hydrogen peroxide [52,53,54]. These radicals inflicted extensive genome-wide DNA mutations, as shown by the generation of R- and M-resistant mutants carrying clinically relevant mutations in the *rpoB* and *gyrA* genes, respectively [52,53]. It is known that Mtb and other pathogens showing a high frequency of mutation-based antibiotic resistance produce ROS-generating enzymes (e.g., NADH dehydrogenase, succinate dehydrogenase and aspartate oxidase), while bacteria with a low mutation frequency lack these enzymes [51].

Many bacteria react to ROS-generated DNA lesions caused by the bactericidal drugs quinolones, aminoglycosides and beta-lactams by inducing the SOS gene regulon, which is regulated by the transcriptional repressor LexA and the protease RecA. In Mtb, we found that treatment with M induced overexpression of SOS genes (e.g., *recA*, *lexA*, *dnaE2*) and DNA repair genes [55]. Induction of the error-prone DNA polymerase dnaE2, which potentially drives increased mutation rates, is considered to be a link between phenotypic persistence and genotypic resistance. Other investigators reported that the M-mediated killing of Mtb involved the accumulation of NADH-dependent ROS, and that the repair of M-mediated lesions and ROS detoxification mechanisms contributed to survival [56,57]. Looking for a therapeutic significance of these observations, they found that Mtb treatment with the respiration stimulator N-acetyl cysteine accelerated respiration, and that ROS production increased M-induced lethality, and lowered the mutant prevention concentration. A study proposed that certain metabolic enzymes of Mtb (e.g., isocitrate lyase) serve antioxidant functions and facilitate drug tolerance [58]. Overall, these observations documented the links among bactericidal antibiotics, ROS production and the formation of drug-tolerant persisters, i.e., of specialized survivors characterized by anoxic metabolism and reduced ATP and ROS synthesis [51].

Low numbers of Mtb persisters are present in the lag and early exponential phases but reach about 1% in the stationary phase [36,59]. Transcriptome analysis of D-cycloserine-formed Mtb persisters showed a distinct pattern of dormancy characterized by a general metabolic downshift and upregulation of a small number of genes overexpressed in other in vitro dormancy models, including the alpha crystallin heat shock protein Acr2 and the sigma factors SigG and SigF. Transcriptome analysis of high persisters (*hip*) Mtb mutants, selected with a lethal dose of R and streptomycin, showed upregulation of some toxin-antitoxins, i.e., the modules in which the toxins lead to growth arrest when labile antitoxins are degraded by proteases [36,60]. *Hip* mutants showed up to 1000 times more persisters than the wild-type strain, a decrease in the phospholipid biosynthesis, and mutations in the cell wall lipid phthiocerol dimycocerosate, an important cell wall lipid and virulence factor. These and other genetic mechanisms suggested that Mtb persisters formation involves multiple pathways, and that *hip* mutants, obtained using both the reference strain H37Rv and human clinical isolates, may contribute to the tolerance of Mtb to drug exposure. Research on Mtb persisters genes will facilitate the development of more effective therapies to eradicate them from TB patients.

## 3. Differentially Detectable (DD) Mtb Cells

Several studies have reported the presence of DD Mtb cells in the sputum produced by patients with active TB. These cells are not able to form colonies on standard glycerol-based solid media (7H10 agar plates and Löwenstein-Jensen slants) but can grow in liquid media supplemented with mycobacterial culture filtrates (CF) [12,13,14,15,16,17,18,61,62] or other substances, such as cyclic-AMP [17] and fatty acids [63,64]. The growth stimulatory effect of CF was ascribed to the five resuscitation-promoting factors (Rpfs) of Mtb, which have the ability to resuscitate non-culturable cells [12,65]. However, the sputum also contains Rpfs-independent DD cells, which do not require Rpfs for growth, and CF-independent DD cells, which do not need CF to resuscitate in liquid media [65]. Thus, the growth stimulatory effect observed in CF is most likely the result of a combination of factors [17].

Dormant DD Mtb cells, which retain stable, low-abundant mRNAs [66], may play a role in disease persistence due to their phenotypic resistance to anti-TB drugs [18,67]. An in vitro DD Mtb cells model was recently developed by Saito et al., allowing the development of ≥90% DD cells after nutrient starvation in a phosphate-buffered saline (PBS) followed by exposure to high R concentrations (PBS-R model) [14]. This model generated DD cells similar to those found in TB patients, and their transcriptional profiles may be useful for monitoring DD populations in the sputum [18].

DD-Mtb cells represented the majority of the bacilli in the sputum of 21% of the patients with DS-TB, and this level increased to 65% after 2 weeks of treatment with first-line drugs [16]. In a study that enrolled subjects with DR-TB (94% MDR patients, i.e., resistant at least to H and R), DD-Mtb cells were found in the sputum of 29% of the patients prior to treatment, and this amount stabilized at 31% after 2 weeks of treatment with a multidrug regimen that included Z, but not R, H and E. However, after 2 months of therapy, DD cells decreased to 13% and 0% in the sputa of DS- and DR-TB, respectively [16]. Another study reported that CF devoid of rpfs yielded a greater amount of DD cells in the sputum of patients with MDR-TB, compared with those with R-monoresistant TB, suggesting that CF is dispensable for the detection of DD cells in DR-strains [65].

In order to explore the biological mechanisms generating DD cells, Saito et al. found that Mtb can enter into a DD state after a sub-lethal oxidative stress damaging DNA, proteins and lipid components [68]. This conclusion was in line with the observations of Hong et al., who reported that ROS formation in response to drugs could prevent *E. coli* cells from growing as CFU, without killing them, and that ROS accumulation mediated by self-amplifying mechanisms continued after antibiotic removal [69]. Transcriptomic analysis showed that, after RIF treatment, DD Mtb cells underwent a partial loss of the oxidative stress response that promoted the formation of the DD phenotype [68]. Thus, Mtb must be able to mitigate oxidative stress to survive in the DD state. However, the capacity of Mtb to do so is limited, so that only intermediate ROS levels lead to DD Mtb. For instance, *Mycobacteriun bovis* and its vaccine derivative, the Bacillus Calmette-Guérin (BCG), are not able to do it, and do not generate DD cells because they succumb to ROS [68]. The observation that the treatment of R-containing cultures in the presence of the antioxidant catalase increased the ability of DD Mtb to form CFU on agar plates reinforced the relationship between oxidative stress and a return to replicative capacity. Hong et al. showed that above a certain host threshold, *E. coli* cells died, but below that threshold, the provisions of anti-oxidants revealed a CFU population [68,69].

In summary, after the treatment of Mtb with bactericidal antibiotics, increasing and self-amplifying ROS levels may be generated. Surviving bacilli may then be detected as CFU (persister cells) if ROS levels are low; LD-MPN (DD cells) if ROS levels are intermediate; or not detected (dead cells) if ROS levels are high. *M. tuberculosis* cells are likely to exist in the host at various levels of oxidative stress and replicability [68]. Overall, these observations indicate that, to eliminate Mtb persisters and DD cells from the host, it would be important to find new drugs/drug combinations inducing oxidative damage under aerobic conditions, and/or other stresses under hypoxic conditions, such as the reactive nitrogen species (RNS) nitric oxide (NO). For instance, NO is induced by pretomanid (Pa, a new anti-TB drug formerly known as PA-824) inside NR Mtb cells [70]. Combinations of Pa with cytochrome bc_1_ inhibitors showed strong bactericidal activity against hypoxic NR Mtb [71].

Finally, besides LD-MPN and CFU counting, in the sputum of TB patients, the presence of a greater mycobacterial load in liquid than in solid media may be detected by the mycobacterial growth indicator tube (MGIT) time to positivity (TTP) assay, a semiquantitative measure of viable Mtb that showed an inverse relationship with the CFU [72,73,74]. The MGIT 960 instrument is an automated system monitoring the fluorescence of an oxygen sensor to detect the growth of mycobacteria in culture tubes containing a modified Middlebrook 7H9 liquid medium, in which the TTP values are reported as days. A study found a strong correlation between the CF^+^ LD-MPN and TTP values for smear-positive and smear-negative sputa [13]. These data indicate that both the LD-MPN and MGIT TTP assays are useful for detecting tubercle bacilli growing on liquid but not on solid media.

## 4. In Vitro Killing of AR+NR Mtb Cells by Drug Combinations

Tools to identify promising in vitro drug regimens for rapid clinical trial evaluation are needed. One such tool is the hollow fibre system model of TB (HFS-TB), a bioreactor containing tubular hollow fibres made of semi-permeable membranes, which showed a 94% predictive accuracy for clinical response rates and optimal exposures [75,76]. In 2015, the European Medicines Agency supported the use of HFS-TB for several drugs, to perform pharmacokinetics-pharmacodynamics monotherapy studies on microbial killing, and to design new combination regimens. After drug exposure, residual viability was evaluated through various tests, including the MGIT TTP assay. Using the HFS-TB model, combinations containing faropenem, linezolid (L) and Z sterilized Mtb cultures in 28 days, as shown by the lack of re-growth of drug-treated cells in MGIT 960 tubes after 56 days of incubation (TTP > 56 days) [77].

A more stringent TTP threshold value was used by our group, who defined the Mtb killing of both AR and NR cells (the latter obtained in the Wayne model) as the lack of regrowth of drug-treated bacilli after 100 days of incubation in MGIT tubes (TTP > 100 days) [74,78,79]. Using this approach, we found that the 4-drug combinations R-M-metronidazole (Mz)-amikacin (Ak) and R-M-Mz-capreomycin (Cp) killed AR+NR Mtb in 21 days [74]. Furthermore, we found that under aerobic and hypoxic acidic conditions (pH of 5.8) likely mimicking those present inside the phagolysosomes of activated macrophages, the 4-drug combinations R-M-Ak-Pa killed AR+NR Mtb in 14 days, while R-M-Ak-nitazoxanide (Nz) killed them in 21 days [78]. Finally, we found that R-Nz-containing combinations, but not R-H-E-Z (currently used for TB therapy), killed NR Mtb in ≥28 days in hypoxia at pH of 7.3, i.e., under conditions likely mimicking those present inside caseous granulomas [79]. Using the MGIT TTP assay, we also reported that the combinations bedaquiline (B)-Ak-rifabutin (Rb)-clarithromycin (Cl)-nimorazole (Nm) and B-Ak-Rb-Cl-Mz-colistin (Cs) killed the AR+NR cells of *Mycobacterium abscessus* in 42 and 56 days, respectively [80]. Overall, our data indicated that the nitro-compounds Mz, Pa, Nz and Nm were important components of combinations killing AR+NR mycobacterial cells. Nitro-compounds are known to induce RNS in anaerobic organisms like *Giardia* spp [81,82] and in NR Mtb [70]. It is possible that combinations containing ROS-inducing drugs like M [52,53,56,57] and R [52,53,54], and RNS-inducing agents like nitro-compounds, are essential for killing the AR and NR stages, which contain drug-tolerant persisters and DD cells.

## 5. Drug Combinations Eradicating Mtb from BALB/c and C3HeB/FeJ Mice

The ability of a drug combination to sterilize TB lesions and prevent relapses due to drug-tolerant persisters and DD cells is determined by the synergistic activities of drug components. In the current regimen used for the treatment of DS TB (R-H-E-Z), R and Z have higher sterilizing activity than H and E [83]. In the last decades, several efforts were made to find new combinations eradicating drug-tolerant bacilli residing in low-vascularized, hypoxic lesions. Such hypoxic conditions are much more present in the caseous granulomas of TB patients and in Mtb-infected caseum-forming animals (e.g., guinea pigs, rabbits, C3HeB/FeJ mice) than in the cellular granulomas of commonly used mice (e.g., Mtb-infected BALB/c mice)—which do not form caseous granulomas, the hallmark of human TB. The guinea pig and rabbit models were not used for the study of combination chemotherapy regimens because of their prohibitive costs [83]. Instead, the C3HeB/FeJ mice, a strain recognized to develop necrotic granulomas mimicking those of human TB, have been used since 2012 for testing the activity of drugs and drug combinations [84,85,86].

However, the low-cost, non caseum-forming BALB/c mice model was the most extensively used for testing drug combinations. In several studies, treatment efficacy was assessed based on lung CFU counts at selected time points during treatment (a measure of bactericidal activity), and on the proportion of mice with culture-positive relapse at least 3 months after the completion of treatment (a measure of sterilizing activity) [83,86,87,88,89,90,91,92,93,94,95,96,97,98,99,100,101,102,103]. Table 1A,B shows a list of 65 combinations sterilizing Mtb H37Rv-infected BALB/c mice.

**Figure 1:**
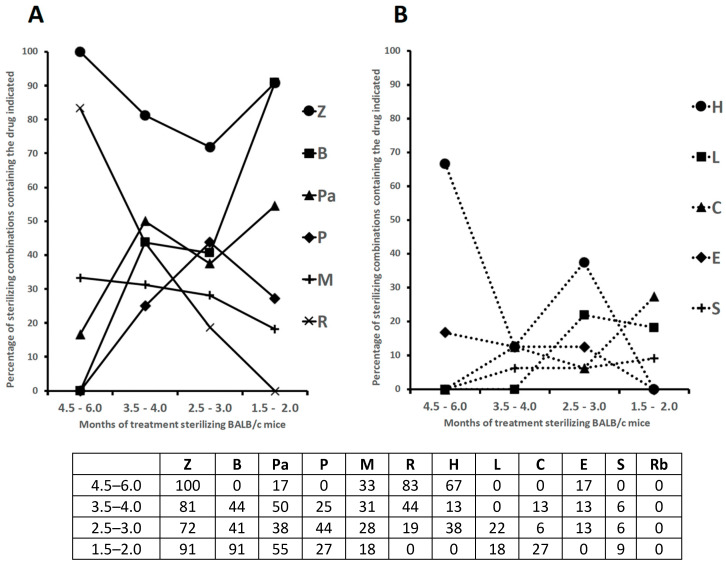
Percentages of sterilizing combinations shown in Table 1A,B, containing the drugs indicated. Data were stratified by the lenght of time required for sterilization. (**A**) Z, pyrazinamide; B, bedaquiline; Pa, pretomanid (PA-824); P, rifapentine; M, moxifloxacin; R, rifampin; (**B**) H, isoniazid; L, linezolid; C, clofazimine; E, ethambutol; S, sutezolid.

A more synthetic view of Table 1 is given in Figure 1A,B, which shows the percentages of sterilizing combinations containing the drugs indicated, which are stratified by monthly periods.

Overall, these data have been useful for identifying pivotal drugs/drug combinations eradicating Mtb from BALB/c mice. Some of these drugs were also tested in combination experiments for the ability to sterilize caseum-forming C3HeB/FeJ mice, and for the efficacy of shortening human TB therapy in well-controlled clinical trials.

### 5.1. Eradication of Mtb from Balb/c Mice

As shown above, Table 1A,B shows a list of 65 combinations, stratified by the minimum length of time required for sterilization (1.5–2.0, 2.5–3.0, 3.5–4.0 and 4.5–6.0 months) [83,86,87,88,89,90,91,92,93,94,95,96,97,98,99,100,101,102,103]. Table 1 also shows information including the months of treatment with each combination, the drug dose in mg/kg of body weight, and some treatment details (5 days/week, etc.) [83,86,87,88,89,90,91,92,93,94,95,96,97,98,99,100,101,102,103]. All the mice were aerosol infected with Mtb H37Rv, with the exception of one study in which animals were infected intravenously [98]. Figure 1A,B shows the percentages of sterilizing combinations reported in Table 1, containing the drug indicated.

The first 6 combinations (regimens 1 to 6) sterilized BALB/c mice after 4.5 to 6 months of treatment [83,91,94,97,99]. Pyrazinamide was contained in 100% of these combinations, followed by R (83%), H (67%), M (33%) and Pa and E (17%); no combinations contained B, L, rifapentine (P), clofazimine (C) and sutezolid (S) (Table 1A). Overall, the administration of R_10_H_25_Z_150_ (regimens 1–3) or R_10_H_10_E_100_Z_150_ (regimen 6) for 2 months, followed by 4 months of R_10_H_25_ (regimens 1–3) or R_10_H_10_ (regimen 6) sterilized the mice in 4.5 to 6 months. These regimens mimicked those currently being used for the treatment of human TB (R-H-E-Z). However, only regimen 6 contained E, a bacteriostatic drug. A 5-month sterilization occurred when H was replaced with M in the intensive and continuation therapy (regimen 4), or when the combination contained the new anti-TB drug Pa (Pa_50_M_100_Z_150_, regimen 5).

Sterilization occurred also after 3.5 to 4 months of treatment with the 16 regimens (regimens 7 to 22) listed in Table 1A. Overall, none contained R_10_H_25_Z_150_ and R_10_H_10_E_100_Z_150_ during the entire period of treatment. In some cases, R, H and Z were administered at high doses. For instance, in regimen 7, after the initial 0.5 months of administration of 25 mg/kg of H (H_25_), the drug was later administered at 75 mg/kg (H_75_). Furthermore, R was administered at 50 mg/kg (R_50_) in regimen 22, and Z at 300 mg/kg (Z_300_) in the intensive phase of regimen 9. A common characteristic of regimens 7–22 was the use of drugs other than R, H and Z, including (i) 15 mg/kg of the long-acting rifamycin P (P_15_) (regimens 7–9) that was substituted for 10 mg/kg of rifampin (R_10_); (ii) 25 mg/kg of B (B_25_, regimens 13–19); (iii) 50 or 100 mg/kg of the nitroimidazole Pa (Pa_50_ or Pa_100_, regimens 10, 12–13, 15–19); iv) 100 mg/kg of M (M_100_, regimens 8–10, 14 and 16); (v) 12.5 or 25 mg/kg of C (C_12.5_, C_25_, regimens 20–21); and (vi) 50 mg/kg of S (S_50_, regimen 15). Overall, Z was contained in 81% of these combinations, followed by Pa (50%), B and R (44%), M (31%), P (25%), H, C and E (13%) and S (6%).

Sterilization was observed also following 2.5 to 3 months of treatment with the 32 regimens (regimens 23 to 54) listed in Table 1A,B. The top 6 drugs used were Z (72%), followed by P (44%), B (41%), Pa and H (38%) and M (28%). Rifapentine was used at 7.5, 10, 15 and 20 mg/kg; Pa was used at 50 or 100 mg/kg.

Finally, the most potent activity was shown by 11 combinations (regimens 55 to 65) that sterilized mice in 1.5–2 months. Ten of them contained both B_25_ and Z_150_, followed by Pa (6/11), P and C (3/11), M and L (2/11) (Table 1B). In this group, the oxazolidinone L, a protein synthesis inhibitor, was used in regimens 64 and 65 and sterilized mice in 1.5 months when combined with B_25_, Z_150_ and Pa_100_.

All these data, reported from 2006 to 2022 mostly by the Center for TB research of the Johns Hopkins University, Baltimore, Maryland, USA, coordinated by Dr. Eric Nuermberger [83,86,87,88,89,90,91,92,93,94,95,96,97,99,100,101,102,103], indicated that all the Mtb H37Rv bacilli (AR cells and NR cells containing drug-tolerant persisters and DD cells) were eradicated from the BALB/c mice, as shown by a lack of relapses (culture-negative lungs) 3 or 6 months after the completion of treatment. Overall, it appeared that the ranking of the top 6 drugs contained in combinations sterilizing the BALB/c mice in 1.5 to 3 months by most regimens (regimens 23 to 65) was Z > B > Pa > P > M > L. It is possible that reactive species, such as ROS (induced by M) and RNS (induced by Pa), contributed to the sterilizing effect. Furthermore, it was evident that the use of R and H sharply decreased as components of the most powerful combinations (Figure 1A,B), and that they were not present in the 1.5- to 2-month sterilizing combinations. Rifampin was replaced with the long-acting rifamycin P [104], which was present in several rapidly sterilizing combinations (Figure 1A). This was likely due to the knowledge that P has a longer half-life than R in human serum (10–15 h versus 2–3 h, respectively) [105].

### 5.2. Eradication of Mtb from C3HeB/FeJ Mice

The results shown in the BALB/c mice were important in designing drug combinations eradicating Mtb in the caseous lesions of C3HeB/FeJ mice, a strain that develops lung necrotic granulomas similar to those found in human TB [84,85]. In 2012, a paper on the dose-ranging comparison of R and P in BALB/c and C3HeB/FeJ mice [86] reported that the long-acting P was roughly four times more potent than the R in both mouse strains.

Table 2 shows a list of 7 combinations published in 2015–2022 that sterilized C3HeB/FeJ mice after 2.5, 3.0, 4.0, 4.5 and 6.0 months [97,102,103].

The administration of R_10_H_10_E_100_Z_150_ for 2 months, followed by R_10_H_10_ for 4 months (regimens 1, corresponding to the first-line therapy of human TB), sterilized the mice in 6 months. The addition of Z at 150 mg/kg (Z_150_) in the continuation phase sterilized the mice in 4.5 months (regimen 2). Thus, continuing Z beyond the first two months was beneficial to prevent relapses in caseum-forming mice. However, this is not applicable to humans, because the use of R-Z beyond 2 months is hepatotoxic, as shown during the prolonged use of this combination for the treatment of LTBI [106]. In regimen 3, the replacement of R_10_ with P_10_, and E_100_ with M_100,_ sterilized the C3HeB/FeJ mice in 4 months [103]. Sterilization was observed after 2.5 to 3 months of treatment with regimens 4–7. In particular, the mice were sterilized after 3 months when R_10_ was replaced with a high dose of P (P_20_), and 12.5 mg/kg of the anti-leprosy drug C (C_12.5_) was added (regimen 4). Clofazimine is a drug that seems in large part to function by inducing ROS in Mtb [102]. The administration of a high dose of the long-acting drug P was important because, in comparison to regimen 1, the mice were sterilized by P_20_H_10_E_100_Z_150_ in 2.5 months (regimen 7).

Finally, due to the knowledge that a strong induction of the cytochrome P450 3A (CYP3A) caused by R and P but not rifabutin (Rb) reduced the concentration of B [107], it was found that the C3HeB/FeJ mice were sterilized after 3 months of B_25_M_100_Z_150_Rb_10_ (regimen 5) or 2 months of B_25_M_100_Z_150_Rb_10_, followed by 1 month of B_25_M_100_Rb_10_, (regimen 6) [103]. Overall, in these 7 regimens, Z was contained in 7 out of the 7 combinations tested, followed by H (5/7), E (4/7), P and M (3/7), B, R and Rb (2/7) and C (1/7). So far, no papers with Pa- and/or L-containing combinations sterilizing C3HeB/FeJ mice were apparently reported.

## 6. New Drug Combinations for Treatment of Human TB

The frequent presence of Z in combinations sterilizing BALB/c and C3HeB/FeJ mice is likely related to the knowledge that this drug penetrates all TB lung lesions and kills persisters residing in caseum [108]. This information makes Z an irreplaceable component of regimens for treating DS-TB [36,109]. In support of this view is that the inclusion of Z in TB treatment shortened the length of therapy from 9–12 months to 6 months [36,109]. Pyrazinamide and R, the two “sterilizing” drugs that contributed the most to treatment shortening, distribute favorably into all lesion compartments, including avascular caseum [110]. However, in the combinations sterilizing the BALB/c and C3HeB/FeJ mice, the use of R progressively decreased in the most rapidly sterilizing combinations because it was replaced by the long-acting rifamycin P (Table 1 and Figure 1A) or the low cytochrome P450 3A-inducer rifamycin Rb (Table 2, regimens 5–6).

Overall, the murine TB data were useful for the choice of drugs to be combined in human TB clinical trials, in order to shorten the current 6-month regimen (R-H-E-Z). This regimen, finalized during the 1980s, and based on seminal studies conducted by the British Medical Research Council in the second half of the 20th century, was widely adopted worldwide for more than four decades. It cured more than 95% of persons, but in some cases long-term adherence to the therapy was difficult to sustain for patients and national resource constraints, contributing to the development of genetic drug-resistance [108,111,112].

Table 3 shows three papers published in the high-impact-factor magazine The New England Journal of Medicine (NEJM), which reported the results of all-oral regimen trials against DS-TB.

All the trials included Z-containing combinations with 6-month [113], 4-month [114] and 2-month [115] efficacies as effective as, or noninferior to, the 6-month control regimen (R-H-E-Z).

The first paper [113] was published in 2014 as a report of the noninferiority trial ISRCTN44153044 (RIFAQUIN trial). It included the control regimen (2 months of intensive therapy with R_600_H_300_Z_1500_E_1200_ and 4 months of continuation therapy with R_600_H_300_) and a 6-month regimen as effective as the control regimen in which H was replaced by a daily dose of 400 mg of M (M_400_) for 2 months, followed by one weekly dose of both M_400_ and a high dose (1200 mg) of P (P_1200_) for 4 months.

The second paper [114] was published in 2021 as a report of the phase 3 noninferiority trial NCT02410772 (Study 31/A5349), and included the control regimen (R_600_H_300_Z_1500_E_1200_), and a 4-month regimen with 2 months (indicated as 8 weeks in the paper) of once-daily P_1200_H_300_Z_1500_M_400_ (R replaced by P, and E replaced by M), followed by 2 months (indicated as 9 weeks in the paper) of once-daily P_1200_H_300_M_400_. This 4-month regimen was supported by the WHO as a possible alternative to the current 6-month regimen for DS-TB, because it showed a similar performance in terms of its efficacy and safety [112,116]. In 2022, the guideline development group of the WHO stated that Study 31 was the first and only phase 3 trial to demonstrate the noninferiority of this 4-month regimen for the treatment of DS-TB when compared to the standard of care [112,116].

The third paper [115] was published in 2023 as a report of the noninferiority trial NCT03474198 (TRUNCATE-TB trial). The authors chose a treatment strategy, rather than focusing on a regimen alone, to be compared with the control regimen [117]. This approach was selected because in clinical trials it is known that at least 85% of participants are cured with 3- and 4-month regimens. Thus, the 6-month standard regimen may lead to overtreatment in the majority of persons in order to prevent a relapse in a minority of persons [115]. Thus, exploring new therapeutic approaches is important. A 5-arms strategy involving initial treatment for 2 months (indicated as 8-weeks in the paper) of R-susceptible TB patients with 5 different regimens was adopted; this was followed up for 96 weeks for the extended treatment of persistent clinical disease and the prompt re-treatment of the minority of persons with a relapse. Briefly, the final results showed that only the 2-month regimen that contained B, L, H, Z and E (B_400/200_L_600_H_300_Z_1500_E_1100_) (Table 3) was noninferior to the 6-month control regimen, with respect to the risk of a composite clinical outcome at week 96.

## 7. Physicochemical Properties of the Drugs Contained in the Sterilizing Combinations

In the framework of the WHO’s vision to eliminate TB as a public health problem by 2035 [1], the three clinical trials published in the NEJM (Table 3) provided robust data towards the realistic possibility of shortening DS-TB treatment using combinations containing Z (found in 3 trials), M, H and E (found in 2 trials), and B, R, P and L (found in 1 trial). The observation that all these drugs were contained in BALB/c and C3HeB/FeJ mice-sterilizing combinations (Table 1 and Table 2 and Figure 1) indicated the usefulness of these small-animal models for testing new TB regimens.

It is difficult to correlate the physicochemical properties of the drugs considered (Table 4), with the in vivo activities shown in the Table 1, Table 2 and Table 3 and Figure 1.

However, some observations may be drawn concerning, for instance, the interplay among the half-life in serum or tissues [118], the unbound fraction (*f*u) (free drug) in caseum or the bactericidal activity in caseum, a lipidic niche for Mtb drug-tolerant persisters and DD cells [42,119,120,121]. In rabbit caseum, the most potent bactericidal drugs were the rifamicins (R, P, Rb: 3.1–4.0 log CFU_max_ decrease in 7 days, Table 4), followed by B, M and Pa (1.6–1.9 log CFU_max_ decrease) [120].

The drugs with half-lives of ≥10 h were, in decreasing order, B (5.5 months) > C (about 25 days) > Rb (45 h) > Pa (18 h) > M (11.5–15.6 h) > P (10–15 h) > Z (9–10 h) [118]. In spite of the small number of studies yet published, Z, B, P, M, Rb and C were major components of the combinations sterilizing the caseum-forming C3HeB/FeJ mice in ≤3 months (Table 3). Of these, Z, B, P, and M were also components of the noninferiority combinations curing DS-TB in ≤6 months (Table 3).

As to the unbound fraction in caseum (*f*u, Table 4), this parameter is inversely related to the lipophilic character of a drug that is mostly expressed as hydrophobicity (logP), i.e., the octanol/water partition coefficient, commonly reported as an experimental or calculated (clogP) value [118]. Drugs with clogP < 0 are considered to be hydrophilic [11]. In Mtb-infected rabbits, only the unbound fraction can penetrate necrotic material or caseum via passive diffusion [119]. The hydrophobicity and aromatic ring count of a drug were shown to be proportional to the caseum binding, and the compounds with clogP < 1 had a high chance of achieving *f*u > 10% [119]. Indeed, the *f*u values of the hydrophilic H and Z (clogP -0.71) (Table 4) were >99.9%, i.e., they could penetrate caseum, while the *f*u of highly lipophilic drugs, such as C and B (clogP of 7.39 and 6.37, respectively) were <0.01%, i.e., they could not penetrate caseum [11,119]. Using a caseum-binding assay and matrix-assisted laser desorption/ionization mass spectrometry (MALDI-MSI) imaging of TB drugs in Mtb-infected rabbits, it was shown that binding to caseum was inversely correlated with passive diffusion into the necrotic core [119]. Among the major positive drivers of binding were high lipophilicity and poor solubility. Thus, highly lipophilic drugs like B and C nonspecifically bind to caseum macromolecules at the outer edge of the caseum core, preventing further passive diffusion toward the center of the necrotic core [119]. Drugs with intermediate clogP values, such as P, R, Pa, and S (clogP of 4.83, 3.85, 2.8 and 1.31, respectively), showed rising *f*u values (*f*u of 0.5, 5.13, 7.31 and 30.1%, respectively). In this view, a rational design of combinations should involve drugs that complement each other in their ability to penetrate caseous granulomas and kill drug-tolerant persisters and DD cells living inside them.

The active combination of the Study 31 trial (P_1200_H_300_Z_1500_M_400_) (Table 3), which cured DS-TB in 4 months, and the combination P_20_H_10_E_100_Z_150_, which sterilized caseum-forming C3HeB/FeJ mice in 2.5 months (Table 2), are quite in keeping with this rationale, because they contain Z, M and P, which are active against persisters and show an unbound fraction (*f*u) in rabbit caseum of >99.9%, 13.5% and 0.5%, respectively (Table 4). However, it is also important to consider the half-life and the extent of Mtb killing in caseum (Table 4) [110,120,121]. For instance, P, which showed a longer half-life than R in human serum (10–15 h versus 2–3 h, respectively) [105, and Table 4], killed Mtb to a similar extent as R and Rb in rabbit caseum after 7 days of incubation [120]. We also found that Mtb was selectively killed by R and P in hypoxia at a pH of 7.3 (the pH of caseum) [44], and others reported that only the rifamycins (P, R, Rb and rifalazil) showed high bactericidal activity in rabbit caseum [120] and fully sterilized it [110]. All these studies indicated that rifamycins may play a pivotal role in eradicating drug-tolerant persisters and DD cells from caseum [110,120,121]. Moreover, the half-lives of M and Z (≥9 h) were in the range of the P half-life value (Table 4). However, it is difficult to explain the sterilizing activity of Z that shortened the length of therapy from 9 months to 6 months but is active only at acidic pH [36,108,109]. Indeed, in rabbit caseum (pH 7.0–7.5), the bactericidal activity of Z was low (0.5 log CFU decrease in 7 days) [110, 121 and Table 4. A possible solution to this enigma was proposed by Sarathy et al. [110], who pointed out that, in closed human granulomas, hypoxia induces the secretion of large amounts of succinate in Mtb. This may generate a “halo” of low pH around the Mtb cells, which favors Z cidality. The observations by Kempker et al. [122], namely that cavity caseum samples obtained from resected lung tissue of 8 out of 10 TB patients had a pH of ≤5.5, are in favor of this hypothesis.

The active combination of the TRUNCATE-TB trial (B_400/200_L_600_H_300_Z_1500_E_1100_) (Table 3) that cured DS-TB in 2 months [115], and the two B-containing combinations (3 months of B_25_M_100_Z_150_Rb_10_, and 2 months of B_25_M_100_Z_150_Rb_10_ followed by 1 month of B_25_M_100_Rb_10_) that sterilized the caseum-forming C3HeB/FeJ mice in 3 months (Table 2, regimens 5 and 6) are more difficult to explain in terms of caseum penetration because B showed a *f*u value of <0.01 in rabbit caseum (Table 4). However, B has a very long terminal elimination half-life (about 5.5 months) [123] due to the great ability of this drug to bind to the intracellular phospholipids and, consequently, to accumulate in tissues. The half-lives of B in the TB lung lesions of the BALB/c and C3HeB/FeJ mice were also high (85.5 and 104.4 h, respectively) [124]. In the C3HeB/FeJ mice, B accumulated within the highly cellular regions in the lungs, but it was also present at reduced but still biologically relevant levels within the central caseum, where it was most likely not detected by the MALDI-MSI system used [124]. Thus, it is possible that the very long half-life of B and/or the synergism with other drugs may contribute to the potent sterilizing activity of B-containing combinations. Some support to this hypothesis comes from the knowledge that, in recent trials, several B-containing combinations were also very active against DR-TB, including patients treated with B-Pa-L for 6 months (ZeNix, trial NCT03086486) [125], with B-Pa-L-M for 6 months (TB-PRACTECAL, trial NCT02589782) [126] and with two other B-containing regimens for 6 or 9 months (STREAM stage 2, trial ISRCTN18148631) [127]. In 2022, taking into account the evidence reported in these and other trials, the WHO published the consolidated guidelines for the treatment of DR-TB [128].

## 8. Conclusions

All the data on drug activity against drug-tolerant Mtb, ranging from the most studied in vitro Wayne dormancy model [40] to the papers on the sterilization of non caseum- [83,86,87,88,89,90,91,92,93,94,95,96,97,98,99,100,101,102,103] and caseum-forming [97,102,103] mice, shed a new light on TB biology [129,130]. These and other preclinical efficacy studies have shown that the greatest difficulties in curing TB do not depend only on intracellular AR bacilli and of their mutants but, above all, on extracellular NR drug-tolerant bacilli living in the caseous tuberculomas [4], the hallmark of TB. Overall, these studies confirmed that the successful treatment of active TB requires the use of drug combinations to eradicate the diverse populations of bacteria present in the infected host [129]. The proportion of non caseum- and caseum-forming mice not exhibiting relapses following different treatments appeared to be useful in selecting candidate drug combinations for clinical evaluation as TB drug regimens [130].

In the last two decades, this new way of seeing the TB boosted the search for novel drugs/drug combinations killing in vitro and in vivo AR cells and NR drug-tolerant cells containing persisters and DD cells. More recently, some of these combinations were tested in several DS- and DR-TB clinical trials, so far achieving in various cases the goal of shortening the duration of the current drug regimens. In 2022, the WHO, following the results of one of these trials, supported the use of a 4-month regimen for the treatment of DS-TB as a possible alternative to the current 6-month regimen [112,116].

## Figures and Tables

**Table 1:** (**A**) Drug combinations sterilizing Mycobacterium tuberculosis H37Rv-infected BALB/c mice (combinations 1–35). (**B**) Drug combinations sterilizing *Mycobacterium tuberculosis* H37Rv-infected BALB/c mice (combinations 36–65).

(A)	Minimum Months of Treatment (Indicated as Grey Boxes) in Which Mice Lungs Were Culture-Negative 3 or 6 Months after Completing Treatment												
Oral Drug Regimens ^a^	1.0	1.5	2.0	2.5	3.0	3.5	4.0	4.5	5.0	5.5	6.0	Treatment Details ^b^	Reference
(1) 2 mo R_10_H_25_Z_150_ + 4 mo R_10_H_25_												5 days/week ^c^	[83]
(2) 2 mo R_10_H_25_Z_150_ + 4 mo R_10_H_25_												5 days/week ^c^	[94]
(3) 2 mo R_10_H_25_Z_150_ + 4 mo R_10_H_25_												5 days/week ^c^	[91]
(4) 2 mo R_10_M_100_Z_150_ + 3 mo R_10_M_100_												5 days/week ^c^	[91]
(5) 5 mo Pa_50_M_100_Z_150_												5 days/week ^c^	[99]
(6) 2 mo R_10_H_10_E_100_Z_150_ + 4 mo R_10_H_10_												5 days/week ^c^	[97]
(7) 0.5 mo R_10_H_25_Z_150_ + 1.5 mo P_15_H_75_Z_300_+ 4 mo P_15_H_75_												2 or 5 days/week ^c^	[88]
(8) 0.5 mo R_10_M_100_Z_150_ + 1.5 mo P_15_M_100_Z_300_ + 4 mo P_15_M_100_												2 or 5 days/week; M: twice/day ^c^	[88]
(9) 2 mo P_15_M_100_Z_300_ + 2 mo P_15_M_100_												2 days/week; M: twice/day ^c^	[89]
(10) 2 mo Pa_100_M_100_Z_150_ + 4 mo Pa_100_M_100_												5 days/week ^c^	[91]
(11) 5 mo R_10_Z_150_												5 days/week ^c^	[92]
(12) 2 mo R_10_Pa_100_Z_150_ + 3 mo R_10_Pa_100_												5 days/week ^c^	[92]
(13) 4 mo B_25_Z_150_Pa_50_												5 days/week ^c^	[87]
(14) 4 mo B_25_Z_150_M_100_												5 days/week ^c^	[87]
(15) 4 mo B_25_S_50_Pa_50_												5 days/week ^c^	[93]
(16) 4 mo B_25_Pa_100_M_100_												5 days/week ^c^	[99]
(17) 4 mo B_25_Pa_100_												5 days/week ^c^	[99]
(18) 2 mo B_25_Pa_100_ Z_150_ + 2 mo B_25_Pa_100_												5 days/week ^c^	[99]
(19) 1 mo B_25_Pa_100_ Z_150_ + 3 mo B_25_Pa_100_												5 days/week ^c^	[99]
(20) 2 mo R_10_H_10_Z_150_E_100_C_12.5_ + 2 mo R_10_H_10_C_12.5_												5 days/week ^d^	[100]
(21) 2 mo R_10_H_10_Z_150_E_100_C_25_ + 2 mo R_10_H_10_C_25_												5 days/week ^d^	[100]
(22) 3.5 mo R_50_H_25_Z_150_												5 days/week ^c^	[98]
(23) 3 mo R_40_H_25_Z_150_												5 days/week ^c^	[86]
(24) 3 mo P_10_H_25_Z_150_												5 days/week ^c^	[86]
(25) 2 mo P_15_M_100_Z_225_ + 1 mo P_15_M_100_												3 days/week; M: twice/day ^c^	[89]
(26) 2 mo P_10_M_100_Z_150_ + 1 mo P_10_M_100_												5 days/week; M: twice/day ^c^	[89]
(27) 2 mo P_7.5_H_25_Z_150_ + 1 mo P_7.5_H_25_												5 days/week ^c^	[90]
(28) 2 mo P_15_H_50_Z_225_ + 1 mo P_15_H_50_												3 days/week ^c^	[90]
(29) 2 mo P_20_H_50_Z_225_ + 1 mo P_20_H_50_												3 days/week ^c^	[90]
(30) 2 mo P_15_M_100_Z_225_ + 1 mo P_15_M_100_												3 days/week ^c^	[90]
(31) 2 mo R_10_Pa_100_Z_150_ + 1 mo R_10_Pa_100_												5 days/week ^c^	[91]
(32) 3 mo R_10_M_100_Pa_100_												5 days/week ^c^	[91]
(33) 4 mo B_25_Z_150_												5 days/week ^c^	[87]
(34) 4 mo B_25_Z_150_L_100_												5 days/week ^c^	[87]
(35) 4 mo B_25_Z_150_R_10_												5 days/week ^c^	[87]
**(B)**													
(36) 3 mo P_10_H_10_Z_150_E_100_												5 days/week ^c^	[86]
(37) 3 mo B_25_S_50_Pa_50_												5 days/week ^c^	[95]
(38) 3 mo B_25_Pa_50_L_100_												5 days/week ^c^	[96]
(39) 1 mo B_25_Pa_100_L_100_ + 2 mo B_25_P_100_												5 days/week ^c^	[96]
(40) 2 mo B_25_Pa_100_L_100_ + 1 mo B_25_P_100_												5 days/week ^c^	[96]
(41) 2 mo B_25_Pa_100_L_100_ + 1 mo B_25_P_100_L_50_												5 days/week ^c^	[96]
(42) 3 mo B_25_Pa_100_L_100_												5 days/week ^c^	[96]
(43) 3 mo B_25_Pa_100_S_50_												5 days/week ^c^	[96]
(44) 1 mo B_25_Pa_100_M_100_ Z_150_ + 2 mo B_25_Pa_100_												5 days/week ^c^	[99]
(45) 1 mo B_25_Pa_100_M_100_ Z_150_ + 2 mo B_25_Pa_100_M_100_												5 days/week ^c^	[99]
(46) 3 mo B_25_Pa_100_L_100_												5 days/week ^c^	[101]
(47) 3 mo R_10_H_10_Z_150_E_100_C_12.5_												5 days/week ^d^	[102]
(48) 3 mo P_20_H_10_Z_150_E_100_												5 days/week ^d^	[102]
(49) 3 mo P_20_H_10_Z_150_E_100_C_12.5_												5 days/week ^d^	[102]
(50) 3 mo P_10_Z_150_M_100_												5 days/week c	[103]
(51) 2 mo R_10_H_10_Z_150_C_25_ + 2 mo R_10_H_10_C_25_												5 days/week ^d^	[100]
(52) 2 mo P_10_H_25_Z_150_ + 1 mo P_10_H_25_												P, H, Z: 5 days/week ^c^	[90]
(53) 2 mo P_10_M_100_Z_150_ + 0.5 mo P_10_M_100_												P, M, Z: 5 days/week ^c^	[90]
(54) 2.5 mo P_20_H_25_Z_150_												5 days/week ^c^	[86]
(55) 3 mo B_25_Z_150_P_10_												5 days/week ^c^	[87]
(56) 4 mo B_25_Z_150_C_20_												5 days/week ^c^	[87]
(57) 2 mo B_25_Z_150_P_10_												5 days/week ^c^	[93]
(58) 2 mo B_25_Z_150_C_20_												5 days/week ^c^	[93]
(59) 2 mo S_50_Pa_50_												5 days/week ^c^	[95]
(60) 3 mo B_25_Pa_100_Z_150_												5 days/week ^c^	[99]
(61) 3 mo B_25_Pa_100_M_100_ Z_150_												5 days/week ^c^	[99]
(62) 2 mo B_25_Pa_100_M_100_Z_150_												5 days/week ^c^	[101]
(63) 1.5 mo B_25_Z_150_P_10_C_20_												5 days/week ^c^	[93]
(64) 1 mo B_25_Z_150_Pa_100_L_100_ + 1 mo B_25_Z_150_Pa_100_												5 days/week ^c^	[96]
(65) 2 mo B_25_Z_150_Pa_100_L_100_												5 days/week ^c^	[96]

(a) R, rifampin; P, rifapentine; H, isoniazid; E, ethambutol; Z, pyrazinamide; B, bedaquiline; M, moxifloxacin; Pa, pretomanid (PA-824); L, linezolid; C, clofazimine; S, sutezolid. Mo, month; dose in mg/kg (subscripted). (b) Drugs were administered once daily by gavage to *M. tuberculosis* H37Rv-infected female BALB/c mice, unless otherwise indicated. (c) Mice were hold for an additional 3 months after completing treatment, and then sacrified to determine the proportion with negative lung cultures (d) Mice were hold for an additional 6 months after completing treatment, and then sacrified to determine the proportion with negative lung cultures.

**Table 2:** Drug combinations sterilizing Mycobacterium tuberculosis H37Rv-infected C3HeB/FeJ mice.

	Minimum Months of Treatment (Indicated as Grey Boxes) in which Mice Lungs Were Culture-Negative 3 or 6 Months after Completing Treatment												
Oral Drug Regimens ^a^	1.0	1.5	2.0	2.5	3.0	3.5	4.0	4.5	5.0	5.5	6.0	Treatment Details ^b^	Reference
(1) 2 mo R_10_H_10_E_100_Z_150_ + 4 mo R_10_H_10_												5 days/week ^c^	[97]
(2) 2 mo R_10_H_10_E_100_Z_150_ + 4 mo R_10_H_10_Z_150_												5 days/week ^c^	[97]
(3) 4 mo P_10_H_10_M_100_Z_150_												5 days/week ^c^	[103]
(4) 3 mo P_20_H_10_E_100_Z_150_C_12.5_												5 days/week ^d^	[102]
(5) 3 mo B_25_M_100_Z_150_Rb_10_												5 days/week ^c^	[103]
(6) 2 mo B_25_M_100_Z_150_Rb_10_ + 1 mo B_25_M_100_Rb_10_												5 days/week ^c^	[103]
(7) 3 mo P_20_H_10_E_100_Z_150_												5 days/week ^d^	[102]

(a) R, rifampin; P, rifapentine; Rb, rifabutin; H, isoniazid; E, ethambutol; Z, pyrazinamide; B, bedaquiline; M, moxifloxacin; L, linezolid. Mo, month; dose in mg/kg (subscripted). (b) Drugs were administered once daily by gavage to *M. tuberculosis* H37Rv-infected female C3HeB/FeJ mice. (c) Mice were hold for an additional 3 months after completing treatment, and then sacrified to determine the proportion with negative lung cultures. (d) Mice were hold for additional 6 months after completing treatment, and then sacrified to determine the proportion with negative lung cultures.

**Table 3:** Oral regimens for treatment of drug-susceptible TB reported to be as effective as, or noninferior to, the control regimen.

	Intensive Phase		Continuation Phase			
Trial Name, Number	Regimen	Administration	Regimen	Administration	Efficiency	Reference
RIFAQUIN, ISRCTN44153044	2 mo R_600_H_300_Z_1500_E_1200_	Daily	4 mo R_600_H_300_	Daily	Control regimen (CR)	[113]
	2 mo R_600_M_400_Z_1500_E_1200_	Daily	4 mo P_1200_M_400_	Once a week	As effective as the CR	
STUDY 31/A5349, NCT02410772	2 mo R_600_H_300_Z_1500_E_1200_	Daily	4 mo R_600_H_300_	Daily	Control regimen (CR)	[114]
	2 mo P_1200_H_300_Z_1500_M_400_	Daily	2 mo P_1200_H_300_M_400_	Daily	Non inferior to the CR	
TRUNCATE-TB, NCT03474198	2 mo R_600_H_300_Z_1600_E_1100_	Daily	4 mo R_600_H_300_	Daily	Control regimen (CR)	[115]
	2 mo B_400/200_L_600_H_300_Z_1500_E_1100_	Daily (*)	_		Non inferior to the CR	

R, rifampin; H, isoniazid; Z, Pyrazinamide; E, ethambutol; M, moxifloxacin; P, rifapentine; B, bedaquiline; Pa, pretomanid; L, linezolid. Mo, month; dose in mg (subscripted). The doses indicated referred to patients of 55–70 kg (Refs. [113,115]) and of ≥55–75 kg (Ref. [114]). (*) Bedaquiline: 400 mg once daily for 2 weeks, then 200 mg three times a week.

**Table 4:** Characteristics of drugs contained in the sterilizing combinations shown in Table 1 and Table 2.

	Pharmacokinetics and		Fraction Unbound (*f*u) and Log CFU_max_ Decrease in Rabbit Caseum	
	Physicochemical Parameters			
Drug, Symbol	Half-Life (h) ^a^	clogP ^b^	*f*u (%) ^c^	Log CFU_max_ Decrease after 7 Days of Incubation ^d^
Rifampin, R	3.35 ± 0.66	3.85	5.13 ± 0.2	3.5
Rifapentine, P	10–15 ^e^	4.83	0.5 ± 0.1	3.1
Rifabutin, Rb	45 ± 17	4.25	n.a. ^f^	4.0
Isoniazid, H	0.5–5	−0.71	>99.9	n.a.
Ethambutol, E	3.3–7	−0.12	35.2 ± 4.4	0.2
Pyrazinamide, Z	9–10	−0.71	>99.9	0.5
Moxifloxacin, M	11.5–15.6	0.01	13.5 ± 3.7	1.8
Linezolid, L	5–7	0.61	29.3 ± 3.6	0.2
Sutezolid, S	n.a.	1.31	30.1 ± 8.7	1.4
Bedaquiline, B	5.5 months	6.37	<0.01	1.9 ^g^
Clofazimine, C	~25 days	7.39	<0.01	0.7
Pretomanid, Pa	18	2.8	7.31 ± 2.2	1.6

(a) Data (± standard deviation) were obtained from DrugBank [118]. (b) clogP predictive values (hydrophobicity) obtained from DrugBank were calculated by ALOGPS [118]. (c) Fraction unbound (fu) data (± SD) in rabbit caseum were obtained from [119]. (d) Log10 CFU max decrease data in rabbit caseum were obtained from [120]. (e) Data were obtained from [105]. (f) Not available, n.a. (g) Log CFUmax decrease after 14 days of incubation [120].

## Data Availability

Not applicable.
